# Development of a multiple-antigen protein fusion vaccine candidate that confers protection against *Bacillus anthracis* and *Yersinia pestis*

**DOI:** 10.1371/journal.pntd.0007644

**Published:** 2019-08-20

**Authors:** Theresa B. Gallagher, Gabriela Mellado-Sanchez, Ana L. Jorgensen, Stephen Moore, James P. Nataro, Marcela F. Pasetti, Les W. Baillie

**Affiliations:** 1 Center for Vaccine Development and Global Health, Department of Pediatrics, University of Maryland School of Medicine, Baltimore, MD, United States of America; 2 BIOMET, University of Maryland School of Medicine, Baltimore, MD, United States of America; 3 Department of Pediatrics, University of Virginia School of Medicine, Box, Charlottesville, VA, United States of America; 4 The Cardiff School of Pharmacy and Pharmaceutical Sciences, Cardiff University, Cardiff, Wales, United Kingdom; Faculty of Science, Ain Shams University (ASU), EGYPT

## Abstract

*Bacillus anthracis* and *Yersinia pestis* are zoonotic bacteria capable of causing severe and sometimes fatal infections in animals and humans. Although considered as diseases of antiquity in industrialized countries due to animal and public health improvements, they remain endemic in vast regions of the world disproportionally affecting the poor. These pathogens also remain a serious threat if deployed in biological warfare. A single vaccine capable of stimulating rapid protection against both pathogens would be an extremely advantageous public health tool. We produced multiple-antigen fusion proteins (MaF1 and MaF2) containing protective regions from *B*. *anthracis* protective antigen (PA) and lethal factor (LF), and from *Y*. *pestis* V antigen (LcrV) and fraction 1 (F1) capsule. The MaF2 sequence was also expressed from a plasmid construct (pDNA-MaF2). Immunogenicity and protective efficacy were investigated in mice following homologous and heterologous prime-boost immunization. Antibody responses were determined by ELISA and anthrax toxin neutralization assay. Vaccine efficacy was determined against lethal challenge with either anthrax toxin or *Y*. *pestis*. Both constructs elicited LcrV and LF-specific serum IgG, and MaF2 elicited toxin-neutralizing antibodies. Immunizations with MaF2 conferred 100% and 88% protection against *Y*. *pestis* and anthrax toxin, respectively. In contrast, pDNA-MaF2 conferred only 63% protection against *Y*. *pestis* and no protection against anthrax toxin challenge. pDNA-MaF2-prime MaF2-boost induced 75% protection against *Y*. *pestis* and 25% protection against anthrax toxin. Protection was increased by the molecular adjuvant CARDif. In conclusion, MaF2 is a promising multi-antigen vaccine candidate against anthrax and plague that warrants further investigation.

## Author summary

Anthrax and plague are ancient infectious diseases that continue to affect people living in poor, endemic regions and to threaten industrialized nations due to their potential use in biowarfare. Candidate vaccines need improvement to minimize non-desirable effects and increase their efficacy. The purpose of this work was to develop and evaluate a single subunit vaccine capable of conferring protection against *Bacillus anthracis* and *Yersinia pestis*. To this end, specific regions from their genome or key protective protein sequences from both microorganisms were combined to obtain either recombinant plasmids or recombinant proteins and tested as vaccine candidates in mice. The recombinant protein MaF2 induced specific antibody responses and afforded full and partial protection against *Y*. *pestis* and *B*. *anthracis*, respectively. Meanwhile, the DNA vaccine equivalent to MaF2 conferred only partial protection against *Y*. *pestis*, which increased when combined with an MaF2 protein boost. MaF2 emerged as a promising dual pathogen recombinant vaccine that warrants further investigation.

## Introduction

*Bacillus anthracis* and *Yersinia pestis* are zoonotic bacteria capable of causing severe and sometimes fatal infections in animals and humans. Although considered as diseases of antiquity in the developed world, they remain endemic in low- and middle-income countries, disproportionately affecting the poor. Even though the threat of natural infection has been markedly reduced in industrialized nations, the same cannot be said for the threat posed by their un-natural use in the context of biowarfare. The ease with which they can be disseminated coupled with high mortality rates, has resulted in their classification as Tier-1 biothreat agents by the US Centers for Disease Control and Prevention (CDC) [1].

*B*. *anthracis*, the etiological agent of anthrax, is a Gram-positive, aerobic, spore-forming bacillus which expresses two major plasmid-encoded virulence factors, a tripartite toxin and an anti-phagocytic capsule. The tripartite toxin is responsible for most of the pathology and comprises a 776-amino acid (aa) metalloprotease called lethal factor (LF), a 767-aa cyclic AMP modulator called edema factor (EF), and a 735-aa non-toxic, cell-binding component called protective antigen (PA), which transports LF and EF into the cell cytosol. PA is the principal protective immunogen in UK- and US-licensed human anthrax vaccines [1–4]. Both of these vaccines require multiple doses to induce protection and because of the manner by which they were developed, they are relatively crude products containing trace amounts of LF, EF, and other bacterial antigens that contribute to the reactogenicity experienced by some individuals [5]. LF and its individual domains have been shown to stimulate a protective antibody response in animals and humans [2, 6, 7].

While it is technically feasible to express and purify individual immunogens, combining the protective regions into a single fusion protein is a more efficient, cost-effective, and practical approach. We have shown that a fusion protein comprising the N-terminal PA binding domain of LF (LFn) and the host-cell-binding C-terminal domain of PA can protect mice against lethal challenge with *B*. *anthracis* [6]. A single vaccine comprising the protective regions from LF and PA would be easier to produce and would confer broader spectrum of protection than one containing PA alone [8].

*Yersinia pestis*, the causative agent of plague, is a Gram-negative bacterium primarily transmitted to humans by the bite of infected fleas, although infection can also occur through direct contact, inhalation, or ingestion of infected materials [9]. Inhalational exposure is a primary concern when considering the use of this organism as a biological weapon because it is often fatal if not treated promptly [10]. Presently, there are no approved vaccines to prevent plague infection. Current vaccines are crude products consisting of either whole-cell formaldehyde-inactivated bacteria or a live-attenuated variant of the pathogen called EV76 [11, 12]. Concerns over their protective efficacy and residual virulence has limited their use and stimulated efforts to develop non-toxic recombinant protein vaccines based on *Y*. *pestis* virulence factors LcrV and F1.

The LcrV antigen is a key regulator of the bacteria’s type III secretion system, which is responsible for the delivery of cytotoxic proteins into the cytosol of mammalian cells [13]. The second vaccine target, F1, is a capsule-like protein that surrounds the bacterium and is thought to inhibit phagocytosis [14]. Passive protection studies in animals using antibodies from humans immunized with a vaccine comprising the F1 and LcrV antigens have confirmed the protective efficacy of these antigens [15]. Vaccination with recombinant F1 [16], LcrV [17] alone or in combination has been shown to protect mice [18, 19] and macaques [20] against plague. Two recombinant protein vaccines based on LcrV and F1 have undergone human trials [21]; they differ in that one comprises a mixture of the LcrV and F1 proteins while the other is a single fusion protein of F1LcrV, which is easier to manufacture.

While these vaccine candidates have been shown to be protective across a range of animal models, they are considered to be suboptimal with regards to the spectrum of antibody responses they generate [22]. For example, the majority of antibodies elicited by PA are non-neutralizing and some have been shown to enhance infection [23, 24]. A similar mixed response has been reported in mice immunized with LFn [23]. This has prompted the investigation of epitope-based vaccines comprising only those regions of PA, LF, and F1 that are key to protection [22]. A single fusion protein consisting of protective regions and immune-stimulatory motifs would induce a rapid and effective immune response, be simpler to produce, stockpile, and administer to populations at risk of exposure to *B*. *anthracis* and *Y*. *pestis* [9, 25]. The clinical evaluation of a one-component vaccine would be simpler and product approval could be expedited.

To develop such a vaccine, a DNA-based approach might also be useful. In addition to simplifying the antigen production process, the DNA platform offers flexibility in manipulation of the vaccine candidate, and the ability to incorporate immunostimulatory components such as cytosine and guanine motifs (CpG) and the RIG (retinoic acid-inducible gene) adaptor protein CARDif (caspase activation and recruitment domain inducing interferon) [26, 27].

In multiple studies, plasmid DNA vaccines have been shown to protect animals against anthrax [28, 29] and plague [30, 31]. However, efforts to develop a multi-agent DNA vaccine against both anthrax and plague have been limited.

Williamson *et al*. demonstrated the feasibility of protecting mice against lethal challenge with *Y*. *pestis* using a prime-boost regimen in which animals were primed with plasmids encoding PA and LcrV, and then boosted with the protein form of LcrV [32]. This immune enhancing effect was confirmed in a subsequent study in which the protective efficacy of a plasmid encoded LcrV-Lfn fusion protein was potentiated by the co-administration of a PA-expressing plasmid [33]. It was postulated that this was due to the presence of immunostimulatory CpG motifs in the backbone of the PA plasmid.

Although popular, CpG motifs rely on expression of its receptor, toll-like receptor 9 (TLR9), which is limited to specialized immune cells [34]. The utility of more ubiquitous immune stimulatory receptors such as RNA helicases has been investigated [27]. Through its interaction with RNA helicase RIG-I, CARDif increases the production of type-I interferon and potentiates immune responses [27].

The aim of this study was to advance the development of a practical, effective, and low-cost, single vaccine formulation against *B*. *anthracis* and *Y*. *pestis* containing only key protective regions. Two constructs consisting of LcrV-LFn fusion supplemented with linear B-cell epitopes from PA and F1 were produced. Immune responses were examined in mice, and the protein candidate MaF2 was selected for further studies. A DNA vaccine encoding MaF2 regions and the molecular adjuvant CARDif was also engineered. Both the multi-antigen fusion protein and DNA vaccine were tested in homologous and heterologous prime-boost combinations. Immune responses and protective efficacy following anthrax and plague challenge were investigated.

## Materials and methods

### Fusion protein design

Genes encoding the PA (Accession AF268967) and LF (Accession M29081) of *B*. *anthracis*, and the V antigen (Accession EFA45641) and F1 capsule (Accession CAB55265) of *Y*. *pestis* were used as templates for the fusion proteins described in this study.

**Fig 1:**
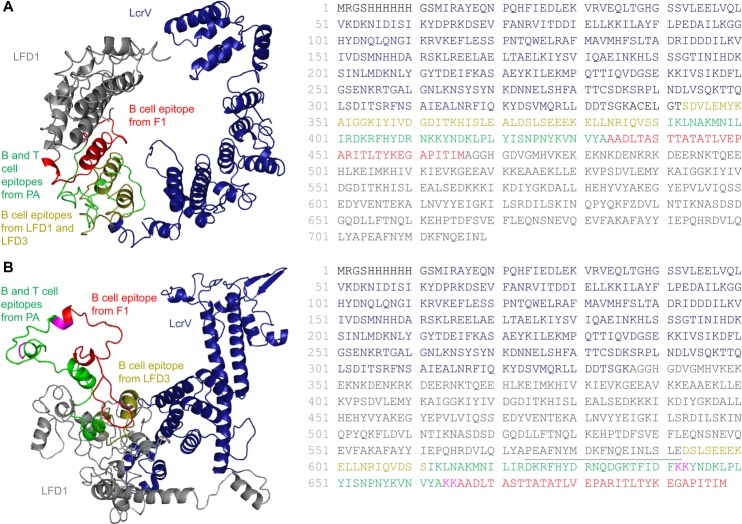
Design and construction of recombinant fusion proteins. Structure prediction was conducted using I-TASSER, and models were selected based on the C-score as calculated by I-TASSER. Ribbon diagrams and amino acid sequences demonstrating the location of individual elements incorporated into fusion proteins. A) MaF1-incorporating antigens from multiple agents; LcrV from *Y*. *pestis* (blue), protective linear B-cell epitopes derived from *B*. *anthracis* LF domains 1 and 3 (yellow), linear B- and T-cell epitopes from within domain 4 of *B*. *anthracis* PA (green), a potential protective linear B-cell epitope from *Y*. *pestis* F1 (red). B) MaF2 contained all elements described for MaF1, except that the PA- and F1-specific epitopes were relocated to the C-terminus region.

A multi-agent fusion protein, LcrV-PA.F1-LFn (MaF1; 719 aa, 82.3 kDa; Fig 1A), was engineered to include the following elements: At the N-terminus, the entire sequence of *Y*. *pestis* LcrV (aa 1–336; shown in dark blue), followed by a linker (ACELGT; aa 337–342). The next region comprised individual protective B-cell linear epitopes from *B*. *anthracis*, LF domain 1 (LFD1) (SDVLEMYKAIGGKIYIVDGDITKHISLEAL; aa 343–372; shown in yellow) and domain 3 (LFD3) (DSLSEEEKELLNRIQVDSS, aa 373–390; also shown in yellow) [35]. The PA element (IKLNAKMNILIRDKRFHYDRNKKYNDKLPLYISNPNYKVNVYA; aa-391-433 shown in green) is a composite of two different regions of PA containing B and T cell epitopes. It has been reported that IKLNAKMNILIRDKRFHYDRN is recognized in part by the murine protective MAbs 2D3, 2D5, 10D2, and 10G4 [36]. It has also been shown that serum from human volunteers immunized with the anthrax vaccine precipitated (AVP) contained antibodies that competed with 2D3 for binding to PA, which suggests that this region is recognized by the human immune system [37]. The small loop region of PAD4 (YNDKLPLYISNPN) is also thought to be the binding site of a number of protective MAbs including 14B7 [6, 37]. An immunodominant CD4 T-cell epitope (YNDKLPLYISNPNYKVNVYA) was identified in PA from aa 682 to 701 [38], and a subsequent experiment that mapped the binding site of a primate toxin-neutralizing antibody also identified a linear epitope within PAD4 (PLYISNPNY); this sequence in PA stretches from aa 686 to 694 [39]. The next element derived from F1 (aa 434–466; shown in red) was a potential protective linear B-cell epitope (AADLTASTTATATLVEPARITLTYKEGAPITIM) identified by mapping the binding sites of a protective murine monoclonal antibody called F104AG1 [40]. The final element was the complete sequence of *B*. *anthracis* LFD1; aa 467–719; shown in grey); together with the LFD1 and LFD3 B epitopes, these elements were added to boost LF-induced immunity. It was also of interest to examine the immune stimulation of the LFD1 and LFD3 B-cell epitopes outside of their natural context (i.e. away from the rest of the LFD1 protein). The LDF1 region has been exploited in multiple studies as a carrier protein to deliver foreign antigens to the immune system, resulting in the stimulation of CD8^+^ and CD4^+^ T cell-mediated immunity [41, 42]. The 3-D structure model, as predicted by I-TASSER, obtained the following scores: C-score of 0.81, exp. TM-score of 0.82±0.08, and exp. RMSD of 6.3±3.9.

A second fusion protein, LcrV-LFnPA.F1 (MaF2; 698 aa, 80.2 kDa), was constructed by relocating the LF-, PA- and F1-specific epitopes to the C-terminus region (Fig 1B). Three terminal aa were added to LFD1 (SLE; aa 590–592), the linear B-cell epitope from LFD1 was removed, while the LFD3 epitope was retained. To enhance peptide processing of the PA-derived sequences, an additional short peptide sequence, QDGKTFIDF, from the small loop of PAD4, a region recognized by several protective PA-specific MAbs was incorporated immediately prior to a dibasic lysine protease cleavage site (KK-aa 664–665; shown in pink) [6, 37, 43]. A second KK site was incorporated at the end of the PA-specific region. The 3-D structure model, as predicted by I-TASSER, obtained the following scores: C-score of -3.25, exp.TM-score of 0.35±0.12, and exp. RMSD: 16.4±3.0.

### Construction and expression of recombinant proteins

Recombinant fusion proteins including PA, biologically inactive LF (LF7, a mutant in which cysteine replaces glutamic acid at position 687), and fusion proteins MaF1 and MaF2 were cloned and expressed from *Escherichia coli* (SG13009 or M15) as recombinant N-terminal histidine-tagged proteins using a commercially available expression system (pQE30 or pQE80L, Qiagen, Inc.). Because of the high AT nucleotide content of the recombinant proteins, the corresponding gene sequences were codon-optimized for expression in *E*. *coli* (GenScript Corp.). Once constructed, all expression vectors were stored at -70°C until required.

Recombinant proteins were produced as previously described [2]. Briefly, recombinant proteins were expressed in *E*. *coli* and purified with Talon metal affinity resin (Clontech Laboratories). Concentrated protein stocks were maintained in HEPES buffer (10 mM HEPES, 50 mM NaCl, pH 7.5) at -20°C. The identities of the proteins were confirmed by SDS-PAGE and western blot analysis (Bio-Rad Laboratories). Recombinant *Y*. *pestis* LcrV and F1 were also produced in *E*. *coli*. Protein bands were detected at the expected size either by staining with Coomassie blue, or after electrophoretic transfer onto nitrocellulose membranes (Bio-Rad Laboratories), using mouse polyclonal antigen-specific sera. The endotoxin content of the different protein preparations was determined by the *Limulus* amoebocyte lysate kinetic-QCL assay according to the manufacturer’s instructions (Lonza). Protein concentrations were determined using the BCA protocol (Pierce, Thermo Scientific).

### Plasmid DNA constructs

The molecular adjuvant (MA) plasmid DNA (pDNA) construct NTC7162-mIPS-1 [pDNA-MA] was derived from the SV40-CMV promoter version of the pDNAVACCUltra plasmid [44] as follows: (1) NE sequence added: this sequence contains a splicing enhancer comprised of SR protein binding site (GAAGAAGA 3x) in exon 2, prior to the start codon for the gene of interest; (2) ISS sequence added: this sequence contains several non-repetitive immunostimulatory CpG motifs; and (3) a DNA fragment encoding the IPS-1 gene was isolated from the plasmid pUNO-IPS-1 and cloned into NTC7162.

The pDNA parental vector, NTC7382-SEAP (pDNA-EV) was derived from the NTC7162 plasmid described above as follows: (1) HTLV-I R-U5 sequence inserted in place of part of CMV intron, for improved eukaryotic expression, (2) Fd gene VIII terminator and tonB bidirectional terminator deleted, for improved *E*. *coli* productivity, (3) *Homo sapiens* placental alkaline phosphatase (SEAP) gene was cloned into the NTC7382 cloning site replacing TPA (native mSEAP secretion sequence used).

**Fig 2:**
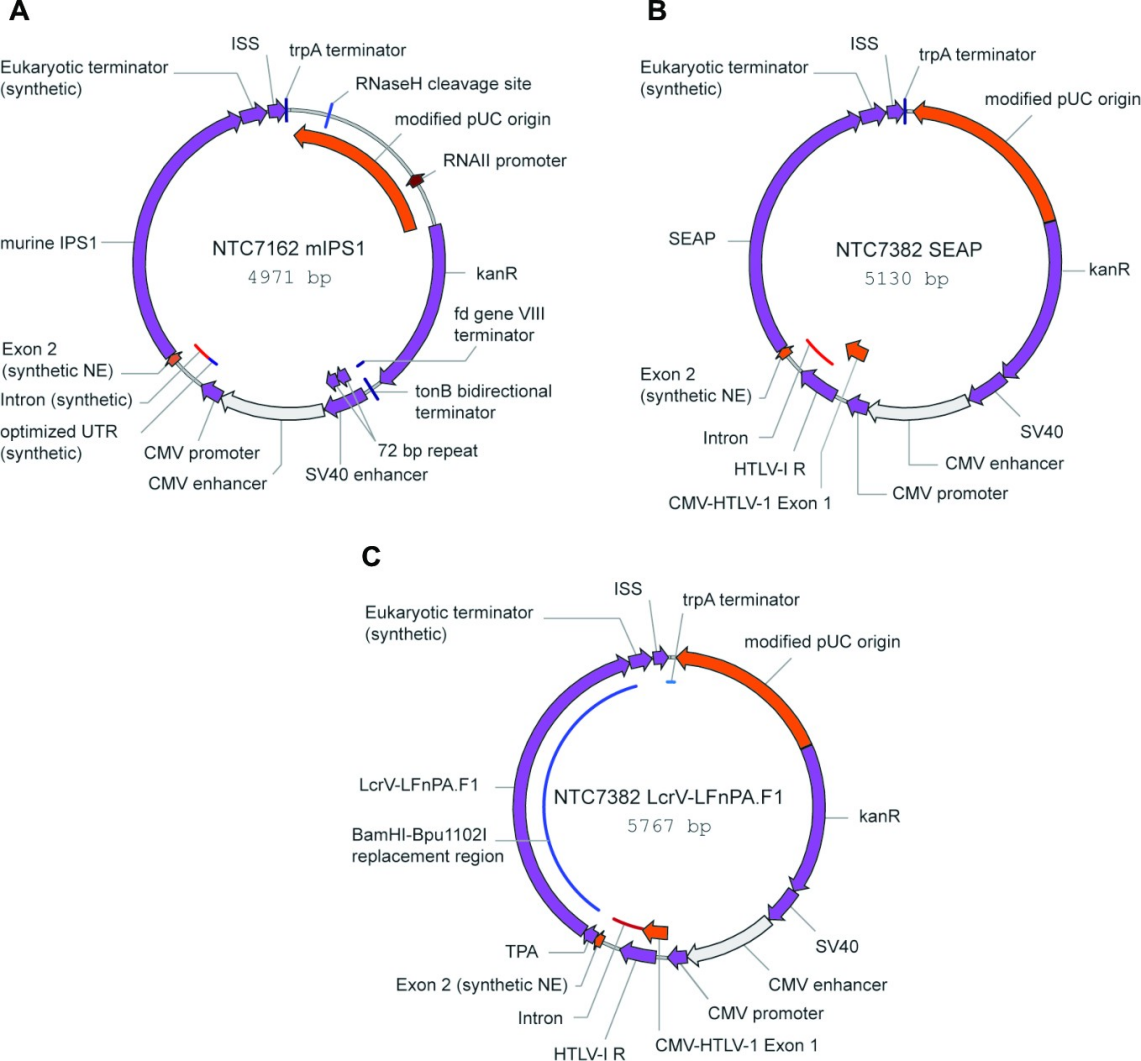
Plasmid DNA construct maps. A) NTC7162 mIPS (pDNA-MA), B) NTC7382 SEAP (pDNA-EV), and C) NTC7382 MaF2 (pDNA-MaF2).

The DNA vaccine NTC7383 LcrV-LFnPA.F1 (pDNA-MaF2) was constructed by transferring the LcrV-LFnPA.F1 gene from the pQE30 expression vector into the NTC7372-HoPaHo vector, replacing the HoPaHo transgene (Fig 2). The 5’ BamHI site was downstream of TPA, so the NTC7382-LcrV-Lfn.PA.F1 construct had the TPA secretion signal upstream and in frame with LcrV-Lfn.PA.F1.

For preparation of stocks of plasmid DNA, CompactPrep Plasmid Giga Kits were used (Qiagen, Inc.). The kit uses a modified alkaline lysis procedure, followed by isolation and purification of plasmid DNA on the silica membrane of the CompactPrep column. Plasmid stocks were resuspended in normal saline (Quality Biologicals Inc.) and stored at -20 ^o^C.

### Mouse immunization and challenges

#### Recombinant protein immunization

Female BALB/c mice (8 to 10 weeks old from Charles River Laboratories) were randomly allocated to different groups (10 per group) and immunized intramuscularly (i.m.) on days 0 and 28 with 10 μg of each of the following proteins: PA, LF, LcrV, F1, MaF1, or MaF2 adsorbed to 25% v/v Alhydrogel (a 100-μL dose was administered as 50 μL in each hind leg). Serum samples were collected on days 0, 13, 27, 42, and 56 after vaccination. Protein-adjuvant adsorption was performed the day before vaccination: the protein was mixed with Alhydrogel (Brenntag Biosector, Denmark) and incubated for 20 minutes at room temperature and then overnight at 4°C.

#### DNA vaccine and protein prime-boost immunization

Groups of 20 female BALB/c mice (8 to 10 weeks old) were immunized as follows (Table 1): (1) for homologous pDNA prime-boost, mice were immunized i.m. with 50 μg pDNA-MaF2 on days 0, 28, and 56 (prime) and again on day 91 (boost); (2) for homologous protein prime-boost, mice were immunized i.m. with 2.5 μg MaF2 plus 25% v/v Alhydrogel on days 0 (prime) and 91 (boost); (3) for heterologous pDNA prime-protein boost, mice were immunized i.m. with either 50 μg pDNA-MaF2, 25 μg pDNA-MaF2 plus 25 μg pDNA-MA, or with 50 μg pDNA-EV (parental vector) on days 0, 28, and 56 (prime) followed by 2.5 μg of MaF2 adsorbed to Alhydrogel at day 91. Unvaccinated mice that received Phosphate-buffered saline (PBS) were also included as controls. All pDNA vaccinations were prepared with 0.25% (v/v) bupivacaine hydrochloride in a final volume of 100 μL PBS [45]. Serum samples were collected on days 0, 27, 55, 84, 105, and 119 after vaccination. Protein-Alhydrogel adsorption was performed the day before vaccination, as described above.

**Table 1:** Evaluation of MaF2 and the MaF2-encoding DNA vaccine in prime-boost immunization.

	Prime			Boost
Immunization	Day 0	Day 28	Day 56	Day 91
DNA/DNA	50 μg pDNA-MaF2	50 μg pDNA-MaF2	50 μg pDNA-MaF2	50 μg pDNA-MaF2
Protein/Protein	2.5 μg MaF2	-	-	2.5 μg MaF2
DNA/Protein	50 μg pDNA-MaF2	50 μg pDNA-MaF2	50 μg pDNA-MaF2	2.5 μg MaF2
DNA+MA/Protein	25 μg pDNA-MaF2	25 μg pDNA-MaF2	25 μg pDNA-MaF2	2.5 μg MaF2
	25 μg pDNA-MA	25 μg pDNA-MA	25 μg pDNA-MA	
Vector/Protein	50 μg pDNA-EV	50 μg pDNA-EV	50 μg pDNA-EV	2.5 μg MaF2
PBS/PBS	PBS	PBS	PBS	PBS

#### *Y*. *pestis* and *B*. *anthracis* challenges

Mice (8 per group) were challenged 31 days after the boost (day 122) with either anthrax lethal toxin, 48 μg of PA and 20 μg of LF (2.5 LD_50_) in 200 μL of PBS, given intravenously (i.v.) [46], or with *Y*. *pestis* EV76 (8 x 10^4^ Colony Forming Units) in 200 μL PBS given i.v. (597 LD_50_) in the presence of FeCl_2_ (40 μg/mouse in 100 μL sterile water intraperitoneally) as previously described [47]. Animals were monitored for survival and signs of disease for 14 days post-challenge. Humane endpoints were strictly observed; any animal that displayed clinical signs indicative of severe infection (e.g., piloerection, posture, dehydration, and mobility problems) was promptly euthanized.

### Antibody responses

Serum IgG specific for *B*. *anthracis* PA and LF and *Y*. *pestis* LcrV and F1 were measured by enzyme-linked immunosorbent assay (ELISA) as previously described [48]. Briefly, plates were coated with LF (1 μg/mL in PBS), PA (2 μg/mL in PBS), F1 (0.5 μg/mL in PBS), or LcrV (0.5 μg/mL in carbonate buffer pH 9.6) for three hours at 37°C. All samples were tested in duplicate, and a positive calibrated control was included in each assay. The avidity of PA-, LF-, and LcrV-specific IgG antibodies was measured by ELISA with an additional 10-minute 6M urea elution step [49]. Avidity index was calculated as the percentage of residual activity (endpoint titer) after treatment with urea. Anthrax toxin-neutralizing activity (TNA) antibodies were measured as previously described [50].

### Antibody-secreting cell (ASC) ELISPOT

The frequency of IgG PA-, LF-, LcrV-, and F1-specific IgG ASC was measured in mice immunized twice with MaF1 and MaF2 fusion proteins as described above (second experiment). Spleens were obtained from 10 mice per group on day 56; The method was performed as previously described [49]. Spots from control wells were subtracted from experimental wells. Results were expressed as mean IgG ASC counts per 10^6^ cells from quadruplicate wells.

### Ethics statement

All animal experiments were approved by the University of Maryland Animal Care and Use Committee under protocol: 0806019. Animal use at University of Maryland at Baltimore complies with the Animal Welfare Act, Public Health Service (PHS) Policy on Humane Care and Use of Laboratory Animals, the Guide for the Care and Use of Laboratory Animals and other applicable regulations, policies, and procedures.

### Statistical analysis

Antibody titers were log-transformed for calculation of geometric mean titer and confidence intervals. For MaF1 immunogenicity experiments, differences in titers between groups at each time point were analyzed with Mann-Whitney Rank Sum Test. For the comparison of homologous and heterologous prime-boost regimes, differences in titers within and between groups were assessed by ANOVA with Bonferroni post-hoc comparison. Unpaired t-tests considering samples with equal variances were performed for avidity indices and IgG ASC responses. TNA titers were compared by Kruskal-Wallis one-way ANOVA with Dunns. A *p-*value of <0.05 was considered statistically significant. Animal survival curves were analyzed using the Gehan-Breslow-Wilcoxon test; for this test a *p*-value of <0.02 was considered statistically significant. Statistical analysis was performed using SigmaStat (Systat Software, Inc.) and GraphPad software (Prism Software).

## Results

### Design and construction of fusion proteins

To construct a vaccine capable of conferring protection against anthrax and plague, two fusion proteins, MaF1 and MaF2, were conceived to include protective regions from both pathogens. The fusion proteins were engineered to allow presentation of these protective epitopes in alternate conformations. MaF1 (719 aa, 82.3 kDa; Fig 1A) contained, at the N-terminus, the entire sequence of *Y*. *pestis* LcrV followed by protective B-cell linear epitopes from *B*. *anthracis* LF domains 1 and 3. Domain 1 of the LF (aa 467–719) was located at the C-terminus. MaF2 (698 aa, 80.2 kDa) contained similar elements but had PA- and F1-specific epitopes relocated to the C-terminus of the fusion protein (Fig 1B); the rearrangement was sought to enhance immune stimulation.

### Design and construction of DNA vaccines

Besides the recombinant proteins, DNA vaccination was used as a platform for immunization with MaF2. Plasmid DNA construct maps are depicted in Fig 2. An MA plasmid (A), parental vector (B), and pDNA-MaF2 (C) were constructed by molecular cloning to be employed as sources of vaccine components.

### Antigenicity of fusion proteins

**Fig 3:**
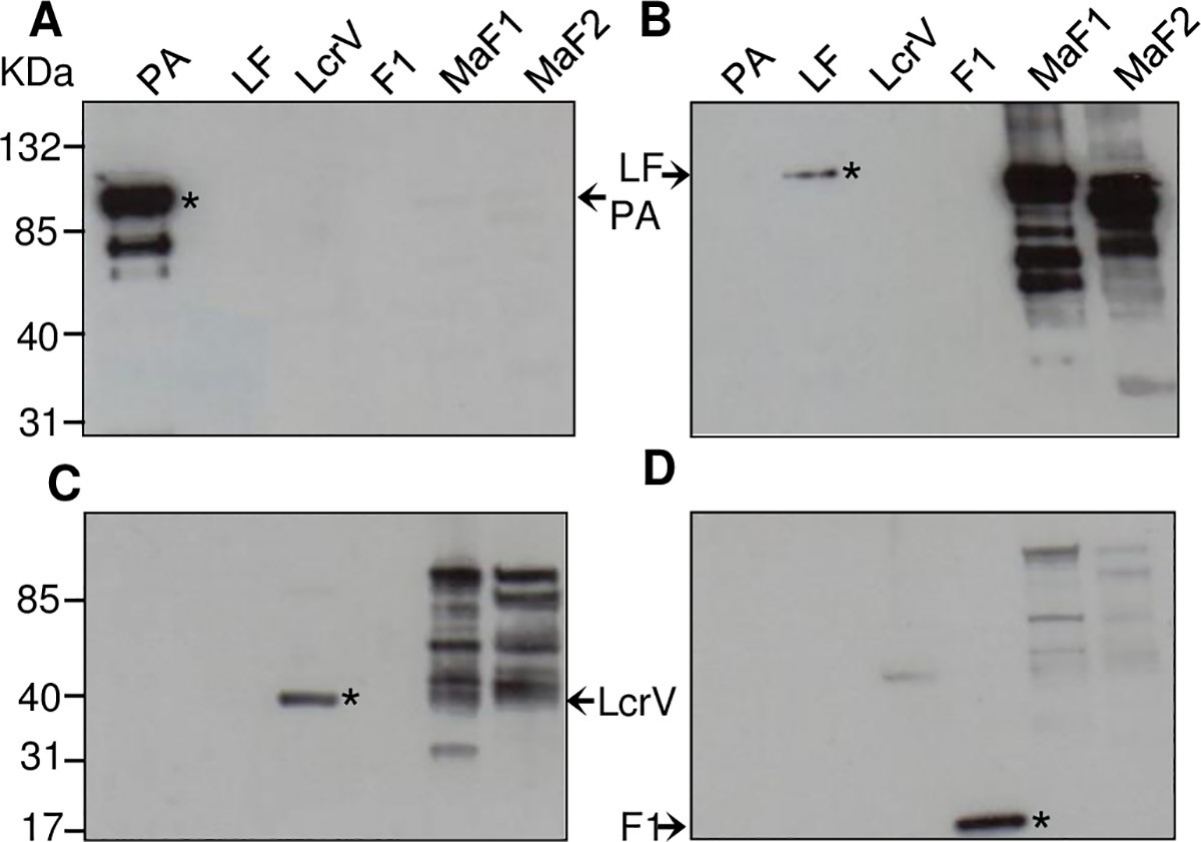
Antigenicity of MaF1 and MaF2 fusion proteins. MaF1 and MaF2 proteins were separated for SDS-PAGE, electrotransferred to nitrocellulose membranes, blocked, and probed with different mouse polyclonal sera: A) anti-PA, B) anti-LF, C) anti-LcrV, and D) anti-F1. Full-length recombinant proteins (*) were used as positive controls. Anti-LF sera contained traces of anti-LcrV IgG.

To confirm the antigenic capacity of each component in the fusion configuration, recombinant proteins were expressed and purified from *E*. *coli*, and analyzed by immunoblot. As expected, antigen-specific murine serum recognized the corresponding full-length PA protein (Fig 3A). Chimeric fusion proteins MaF1 and MaF2 were clearly recognized by anti-LF and anti-LcrV antisera (Fig 3B and 3C). Even though a degradation pattern was detected, the more evident bands were at the expected molecular size (~82.3 kDa for MaF1 and ~80.2 kDa for MaF2). Although robust signals were seen against LcrV and LF, this was not the case for PA and F1. The strongest reactions to each fusion protein were seen against LcrV and LF using specific antisera, which was expected given that these regions comprised a substantial part of each fusion protein. In contrast, the PA- and F1-specific regions were engineered into the protein as much smaller fragments (PA: 44 aa; F1: 32 aa), which may partially explain the lack of recognition by PA- and F1-specific antisera (Fig 3A and 3D). Another possibility is that cryptic epitopes of PA and F1 were formed.

### Immunogenicity of the MaF1 and MaF2 fusion proteins

**Fig 4:**
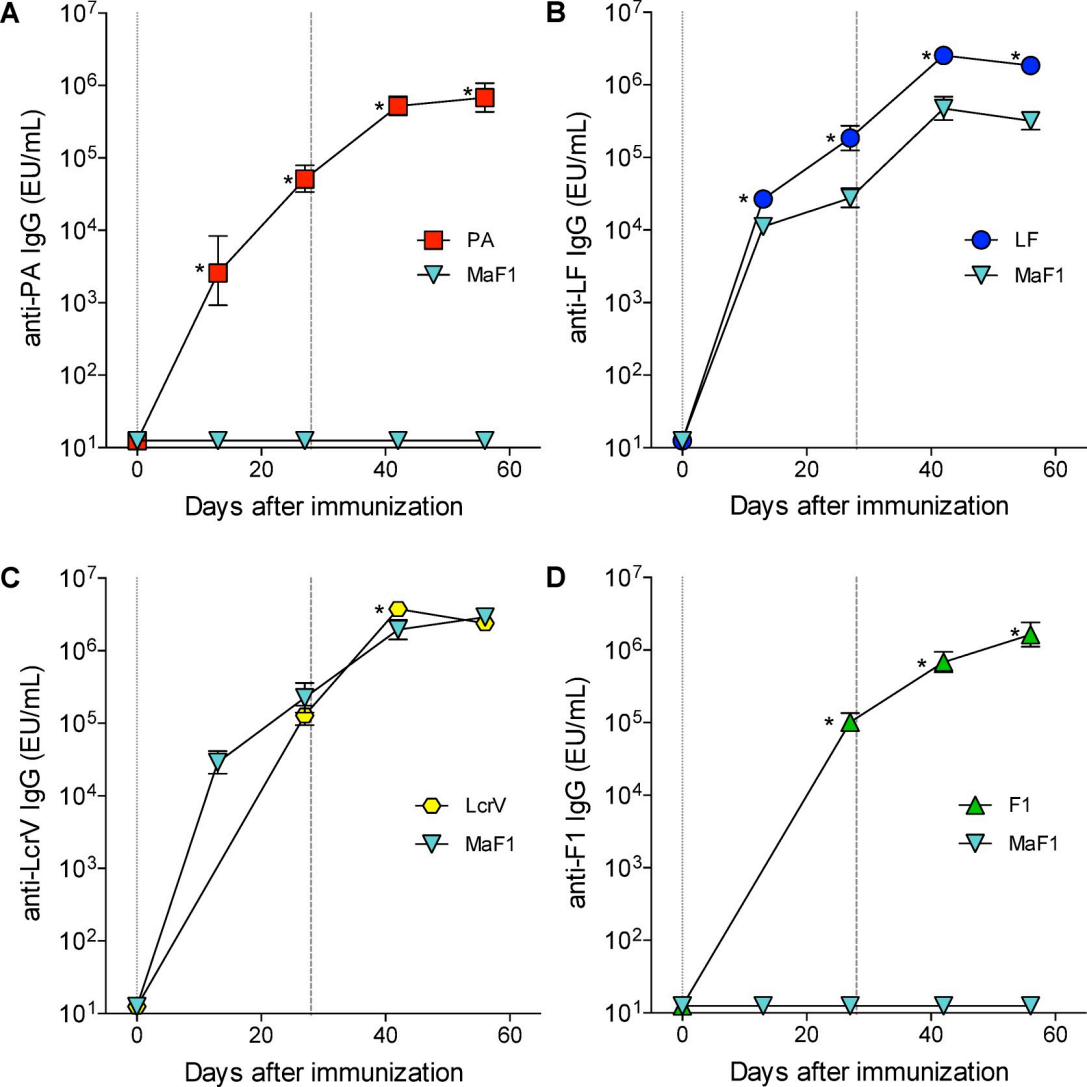
Kinetics of antibody responses elicited by fusion protein MaF1 and full-length proteins. A) PA-, B) LF-, C) LcrV-, and D) F1-specific serum IgG titers. Mice (10 per group) were immunized i.m. with 10 μg of each protein plus alum. Vertical lines indicate days of immunization, dotted lines represent prime (day 0), dashed lines represent boost (day 28). Data represent geometric mean titers and ± 95% confidence intervals. * indicates significant differences between groups (*p*<0.05).

In a first attempt to assess immunogenicity of the fusion proteins, mice were immunized i.m. with 10 μg of PA, LF, LcrV, F1, or MaF1 proteins twice, 28 days apart, and the kinetics of antigen-specific serum IgG responses were determined by ELISA. Mice immunized with the MaF1 fusion mounted a strong LcrV-specific IgG response similar to that seen in mice immunized with full-length LcrV (Fig 4C). This was expected because the fusion protein was engineered to incorporate the whole sequence from LcrV at the N-terminal. The MaF1-immunized mice also mounted a robust LF-specific IgG response although somewhat lower than that seen with full-length LF (Fig 4B), presumably because only the N-terminal region of LF was included in the fusion protein. Antibody responses to LF and LcrV further increased after the MaF1 boost at day 28 (Fig 4B and 4C). In contrast, MaF1 failed to induce serum IgG responses to PA and F1 (Fig 4A and 4D); this observation agrees with the lack of PA and F1 signals in the immunoblot analysis.

**Fig 5:**
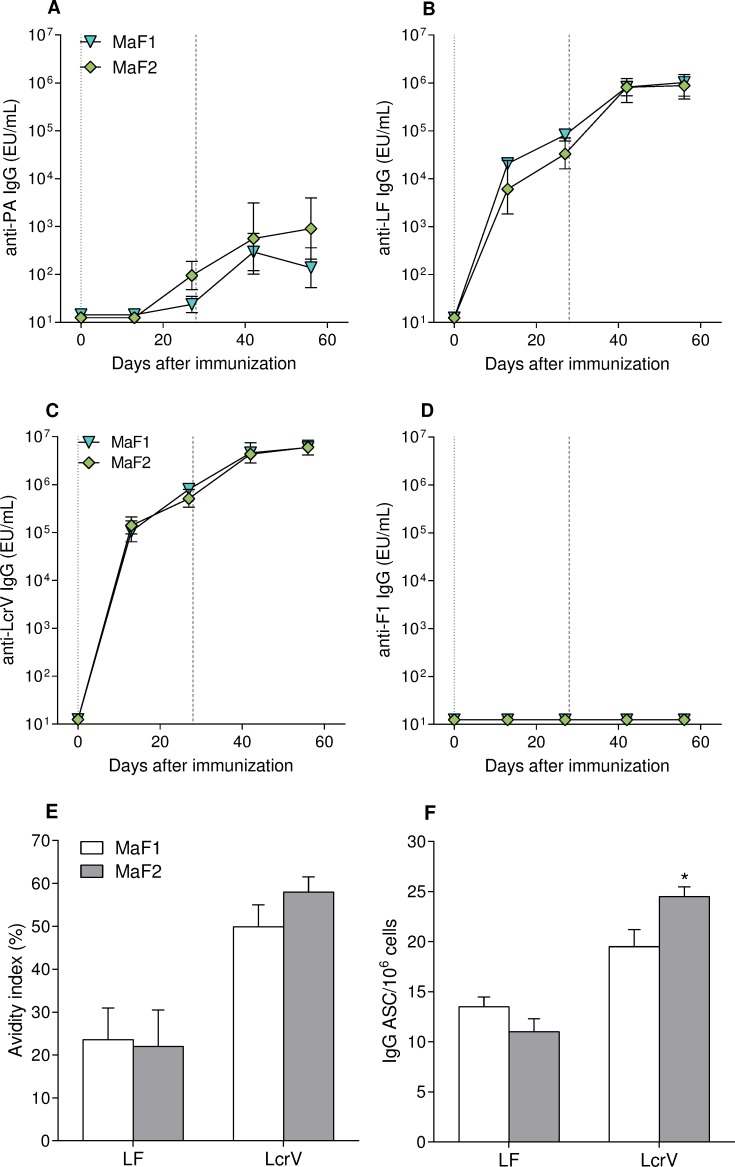
Antibody responses elicited by multi-agent MaF1 and MaF2 fusion proteins. A) PA-, B) LF-, C) LcrV-, and D) F1-specific IgG titers. Mice (10 per group) were immunized i.m. with 10 μg of each protein plus alum. Vertical lines indicate days of immunization, dotted lines represent prime (day 0), dashed lines represent boost (day 28). Data are shown as geometric mean titers ± 95% confidence intervals. E) Avidity index of LF- and LcrV-specific serum IgG at day 56. Results represent mean avidity index± SE from 10 mice per group. F) LF- and LcrV-specific IgG spleen ASC measured on day 56. Results are shown as mean IgG ASC per 1×10^6^ cells ± SE of replicate wells. * indicates significant difference (*p*<0.05) compared to MaF1.

In a second study, the serum IgG responses induced by MaF1 and MaF2 were compared to determine whether the relocation of the PA- and F1-derived sequences to the C-terminus of the protein in MaF2 would enhance host immunity to these antigens. Like MaF1, immunization with MaF2 resulted in high (and similar) levels of LF- and LcrV- specific serum IgG (Fig 5B and 5C), and these antibodies exhibited similar avidity index (Fig 5E). Mice immunized with either fusion protein also developed LF- and LcrV-specific systemic IgG ASC (indicative of vaccine-induced functional B cells that can replenish plasma cells in circulation), although the frequency of LcrV-IgG ASC was higher in the MaF2-immunized group (Fig 5F). In this experiment, mice immunized with MaF1 and MaF2 had detectable, although low, PA-specific serum IgG responses while no responses were seen against F1 (Fig 5A and 5D). These results suggest that the relocation of the PA sequences had little effect on immunogenicity and that the F1-derived sequences, as incorporated in the fusion proteins, did not seem to engage the murine immune system. Given the fact the MaF1 failed to stimulate a detectable PA antibody response in the first experiment and elicited somewhat lower PA IgG titers as compared to MaF2 in the second experiment, MaF2 was selected for further studies.

### Immunogenicity of pDNA-MaF2 and MaF2 homologous prime-boost immunization

To compare the immunogenic capacity of the DNA vs the recombinant multi-fusion protein in a homologous prime-boost regimen, mice were immunized with 50 μg pDNA-MaF2 or with 2.5 μg of MaF2-alum (Fig 2). Mice were primed with MaF2 on day 0 and boosted on day 91. The groups that received pDNA-MaF2 were primed on days 0, 28, and 56 (because pDNA is known to be less immunogenic than protein) and boosted on day 91 (Table 1).

**Fig 6:**
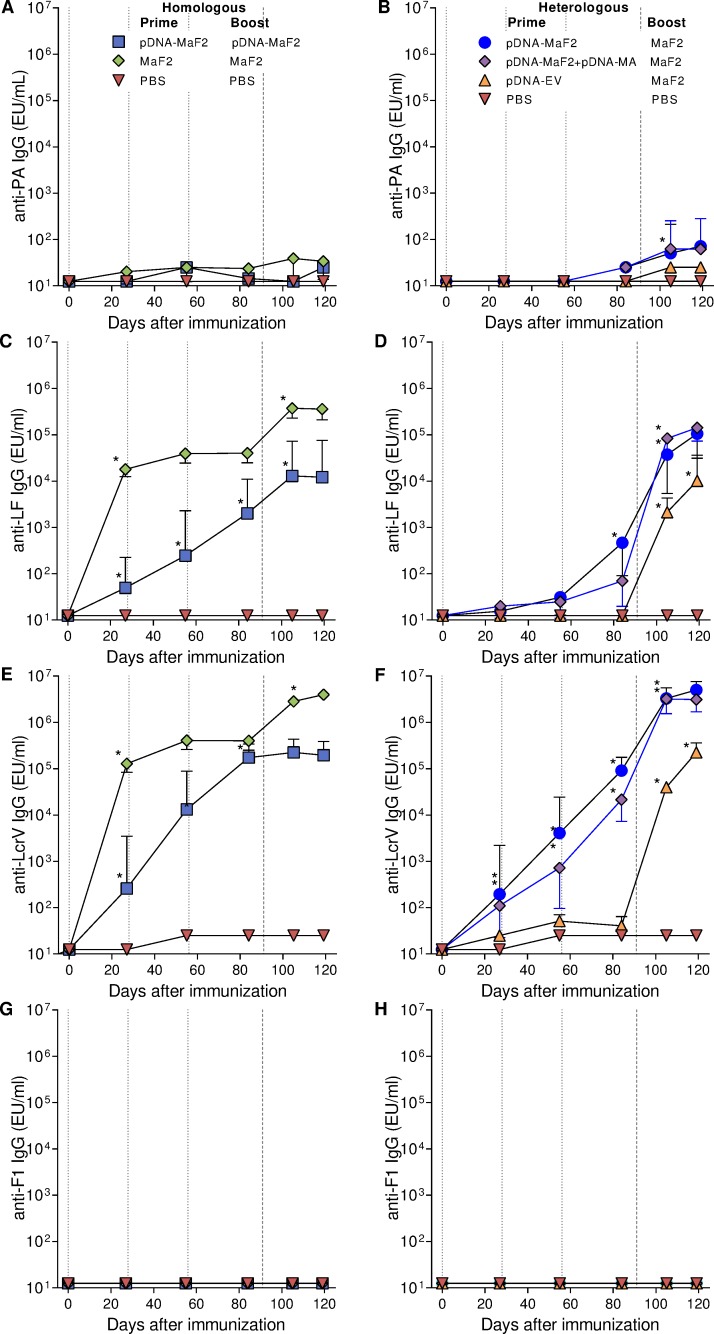
Antibody responses of mice immunized with a homologous and a heterologous prime-boost immunization schedule. A-B) PA-, C-D) LF-, E-F) LcrV-, and G-H) F1-specific serum IgG titers. Mice (20 per group) were primed on days 0, 28, and 56 with pDNA vaccine constructs or on day 0 with MaF2; all groups were boosted on day 91 (see Table 1). Dotted lines indicate prime immunizations and dashed lines represent boost. Data represent geometric mean titers and 95% confidence intervals. * indicates a significant increase in titer (*p*<00.5) compared with the previous time point.

Both the pDNA-MaF2 and MaF2 vaccines stimulated LF- and LcrV-specific serum IgG (Fig 6C and 6E), although significantly higher titers were produced by the group that received MaF2 (*p*<0.001). The fusion protein elicited prompt and robust IgG responses that persisted for up to three months and further increased after the boost. In contrast, DNA vaccination resulted in lower responses despite multiple priming doses. Different from LF and LcrV responses described above, both MaF2 and pDNA-MaF2 failed to stimulate detectable PA- or F1-specific IgG (Fig 6A and 6G).

### Immunogenicity of pDNA-MaF2 and MaF2 fusion protein heterologous prime-boost regime

We next compared antibody responses produced by pDNA-MaF2 prime followed by MaF2 boost in a heterologous prime-boost regimen. Mice received pDNA-MaF2 as described above; additional groups received pDNA-MaF2 admixed with an MA or pDNA-EV as control for priming. All were boosted with MaF2. An unvaccinated (PBS) group served as negative control. Serum IgG responses to F1 were again negligible (Fig 6H), while a marginal increase in PA-specific IgG was detected in the pDNA-MaF-primed MaF2-boosted groups (Fig 6B).

The LF- and LcrV-specific IgG responses greatly improved in the pDNA-MaF2-primed mice following the MaF2 boost, with titers surpassing those of unprimed controls (parental vector). The overall kinetics of IgG production for both antigens was similar among the groups (Fig 6D and 6F). In both the homologous and heterologous prime-boost experiments, IgG responses to LcrV were faster and of higher magnitude than those against LF.

The molecular adjuvant (pDNA-MA) did not improve antibody responses induced by pDNA-MaF2.

### Anthrax toxin neutralizing responses

**Fig 7:**
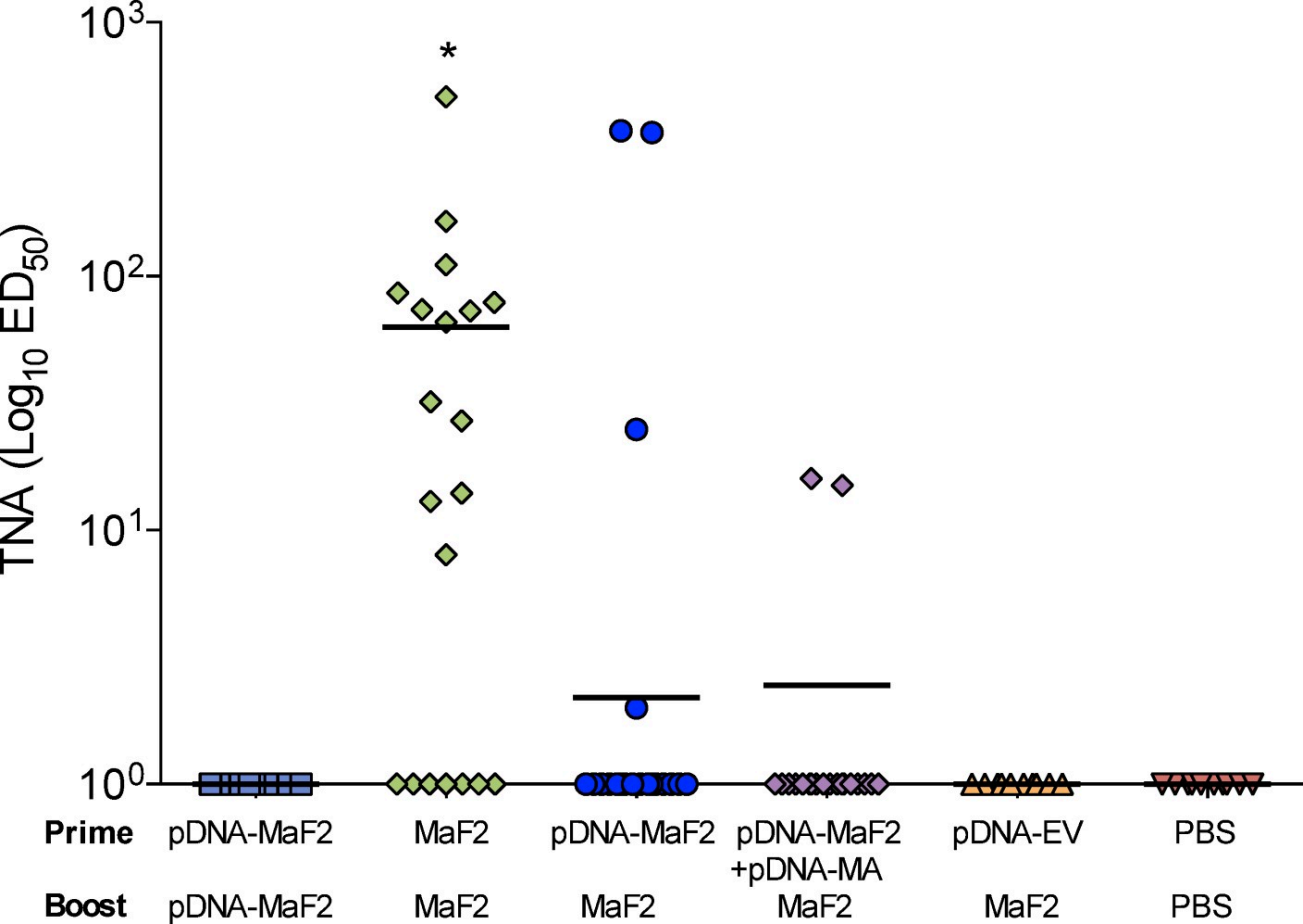
Toxin-neutralizing activity titers induced by and pDNA-MaF2 and MaF2 prime-boost immunization. Mice were immunized as described in Table 1. Data represent individual TNA titers and geometric mean from 10‒20 mice per group. * indicates a significant difference (*p*<0.05) compared to other treatments.

The ability to generate antibodies capable of neutralizing the activity of the lethal toxin of *B*. *anthracis* has been associated with protective efficacy of anthrax vaccines in animal studies [51, 52] and serum toxin-neutralizing antibody activity is an accepted correlate of protection for purposes of anthrax vaccine development. Importantly, despite the low PA-specific IgG levels detected by ELISA, mice immunized with MaF2 had elevated serum anthrax toxin-neutralizing antibodies, with titers surpassing those of all other groups (Fig 7).

### Protection against challenge with *B*. *anthracis* lethal toxin and *Y*. *pestis*

To determine protective capacity of the various immunization regimens against *B*. *anthracis* lethal toxin and *Y*. *pestis* lethal infection, mice immunized as described above were challenged 31 days after the last immunization with either anthrax lethal toxin i.v. (2.5 LD_50_) or *Y*. *pestis* EV76 i.v. (597 LD_50_) supplemented with iron (to increase virulence).

**Fig 8:**
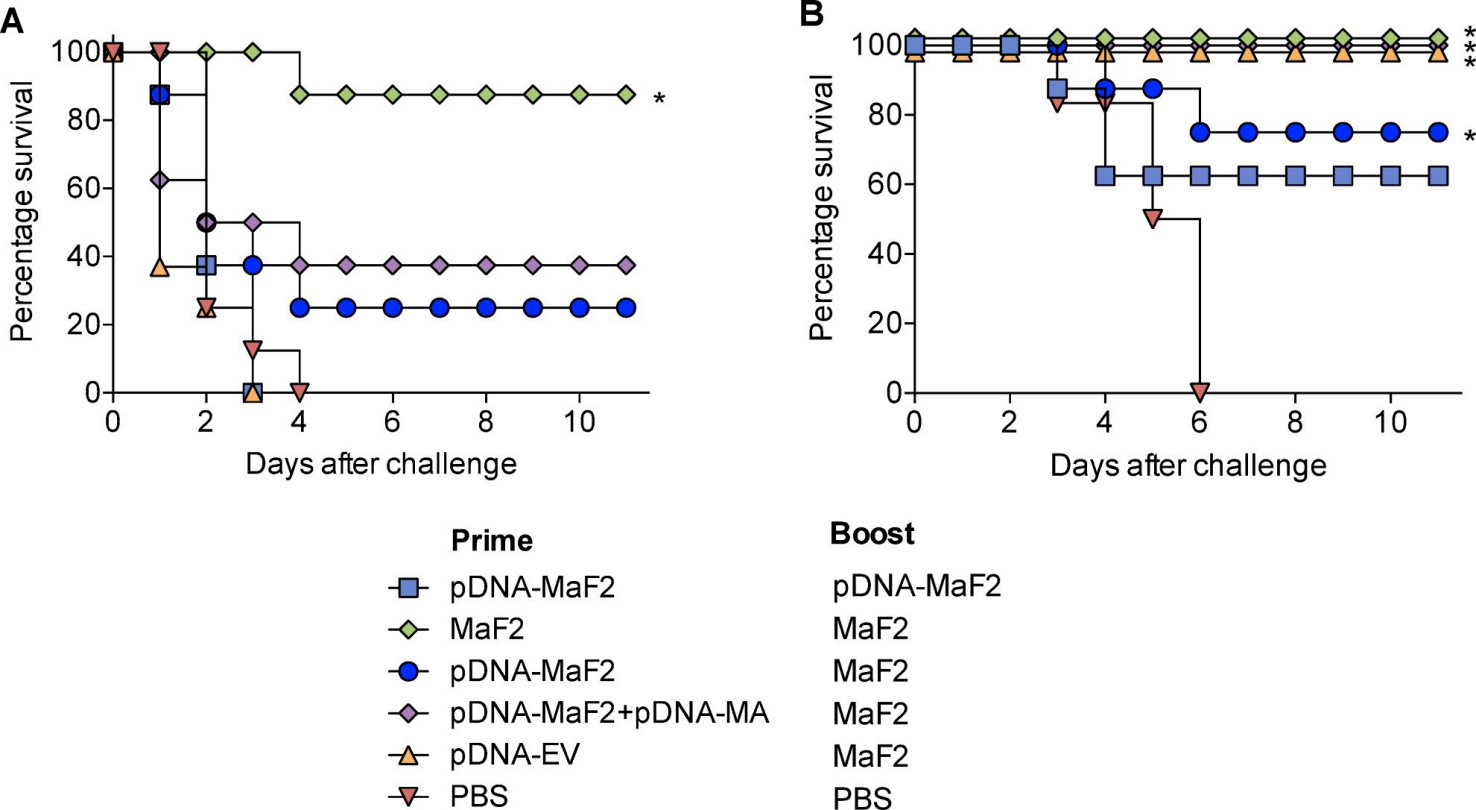
Survival rates of pDNA-MaF2 and MaF2 immunized mice following anthrax lethal toxin and *Y*. *pestis* lethal challenge. Mice were immunized as described in Table 1 and challenged 31 days post boost with A) anthrax lethal toxin or B) *Y*. *pestis* EV76 plus iron. Curves represent survival rates from 8 mice per group; * indicates significant difference (*p*<0.02) compared with unvaccinated (PBS) group.

Homologous MaF2 prime-boost immunization conferred the highest degree of protection (88%) against lethal anthrax toxin challenge, which was in agreement with the high toxin-neutralizing titers in this group (superior to all other treatments). In contrast, modest protection (<40%) was observed in mice primed with pDNA-MaF2 and boosted with MaF-2 (Fig 8A).

The MaF2 prime-boost conferred complete protection against *Y*. *pestis* lethal challenge (Fig 8B). Similarly, a high level of protection (75%) was attained by pDNA-MaF2-prime followed by MaF2-boost. Interestingly, mice primed with pDNA-MaF2 and the MA, and even mice primed with the parental vector, achieved complete protection after the MaF2 boost, which suggests that protective immunity could be attained with a single MaF2 immunization. Among mice primed and boosted with pDNA-MaF2, 63% survived the *Y*. *pestis* challenge prime-boost although this survival rate did not reach statistical significance (*p* = 0.26) compared to the unvaccinated (PBS) control.

## Discussion

Prior immunization of individuals would greatly reduce the impact of both a natural outbreak and a bio-terror attack using either *Y*. *pestis* or *B*. *anthracis*. Intentional exposure of unprotected civilians to these organisms would cause many casualties and major socio-economic disruption. Therefore, safe and effective alternatives for treatment and prophylaxis for prevention of these two neglected diseases remains a high public-health priority.

A major goal of anthrax vaccine manufacturers has been to develop non-toxic recombinant protein vaccines based on PA [1, 53]. Although these next-generation products are likely to elicit fewer side effects [22], there are concerns that they may stimulate a less robust immune response and thus afford less protection. A vaccine containing both PA and detoxified LF would confer broad protection, particularly against strains of *B*. *anthracis* in which PA has been genetically modified, either by nature or by man [54].

Similar to PA for anthrax, LcrV + F1 are considered the main vaccine targets for a plague vaccine due to their capacity to elicit protective immunity in a variety of model animal species. However, the spectrum of antibody responses generated by these antigens in humans may not be optimal [8, 22, 55]. A region of LcrV (amino acids 271–300) have been shown to suppress the host immune response while F1 tends to form heterogeneous aggregates, which might negatively affect the quality of the vaccine and ensuing immune responses [56, 57].

For rapid mass immunization programs, as would be required in the face of a bioterrorist attack, the use of multiple vaccines is impractical because of cost and logistical challenges. A multi-agent vaccine capable of conferring protection following a single dose would be more cost effective and easier to implement. This approach has been tested by combining separate recombinant proteins from both microorganisms, and it has been shown that PA, F1, and LcrV can be successfully co-delivered without decreasing their protective efficacy [25]. A recent study confirmed the feasibility of a two-dose immunization with a fusion protein comprising full length F1, LcrV, and PA combined with alhydrogel for protection of mice, rats, and rabbits against lethal challenge with *B*. *anthracis* and *Y*. *pestis* [52].

Thus, we investigated the feasibility of developing a combined vaccine capable of conferring protection against both plague and anthrax in a single formulation and potentially using a single immunization. To eliminate regions that might decrease the robustness of the immune response, we constructed fusion proteins based on full-length LcrV, the N-terminal region of LFn, and individual epitopes derived from PA and F1.

A single dose of the fusion protein MaF2 stimulated complete protection against *Y*. *pestis* (Fig 8B), which could be attributed to the production of high levels of LcrV-specific IgG. Two doses of the fusion protein also induced a high level of protection against anthrax lethal toxin. The efficacy of MaF2 with a 3-month delayed boost is noteworthy and appealing, as such a vaccine could be useful for priming high-risk individuals who could be boosted (ensuing rapid anamnestic response) later, if needed. Although an increase in PA antibody titers was observed after boosting, the contribution of this response to protection is unclear, given that LF-IgG titers were higher and LF is also able to stimulate the production of toxin-neutralizing antibodies.

In the context of the PA-specific response, this result was disappointing, given that the prime reason for employing an epitope-based approach was to eliminate those regions of PA that subtracted from the quality of the protective immune response. The sequences used were identified by monoclonal antibody mapping studies. The PA-derived CD4 T-cell epitope, on the other hand, although immunodominant for humans, might not have been recognized by the mice used in this study [38].

We next sought to determine whether similar levels of protection could be achieved when MaF2 was expressed from a DNA vaccine. Different from the recombinant proteins, DNA vaccines are simple to design and engineer, and can incorporate multiple vaccine targets, as well as immune-stimulatory sequences and regulators, into a single vector. They can be freeze-dried, making them more cost-effective to stockpile, and can be delivered using needle-free approaches, reducing the logistical burden of immunizing large numbers of people during a natural outbreak or a bioterrorist threat [58, 59].

The group primed with pDNA-MFa2 and boosted with MaF2 exhibited substantial protection against *Y*. *pestis*. The presence of the MA CARDif and the parental plasmid vector during the priming immunization seemed to have enhanced survival post challenge, suggesting that the DNA itself may have immunostimulatory properties. While the homologous pDNA-MaF2 prime-boost immunization elicited some protection against *Y*. *pestis*, the survival rate was not statistically significantly different from that of the PBS control, indicating that further work would be needed to enhance the immunogenicity of the DNA vaccine. In contrast to plague, the pDNA-MaF2 prime-boost resulted in only partial and non-significant protection against anthrax toxin, confirming that the immunogenicity of this construct was not optimal. Interventions that could improve immunogenicity include the use of other routes of vaccination (i.e. intradermal) and the incorporation of additional immune stimulatory factors, such as CpG motifs, into the DNA vector backbone. The addition of an immunostimulatory oligodeoxynucleotide compound (CpG 7909) as an adjuvant to enhance the immunogenicity of BioThrax, is currently being investigated in humans [60].

In conclusion, the MaF2 fusion protein conferred complete (100%) protection against *Y*. *pestis*, and high levels of protection (88%) against anthrax toxin in mice. Work is in progress to enhance the efficacy of the *B*. *anthracis*-derived elements, to reduce the size of the LcrV region, and to enhance the F1 specific response. The protective efficacy of these optimized constructs will be examined in more complex challenge systems involving other forms of disease. The experimental evidence obtained will facilitate its evaluation in human clinical studies. The creation of a single dose vaccine capable of being stockpiled and of stimulating rapid protection in the event of a covert biological attack would markedly reduce the impact of such a tragic event. Indeed, such a resource would be a highly valuable public health tool to protect at-risk populations around the world.
